# MPMR: Multi-Scale Feature and Probability Map for Melanoma Recognition

**DOI:** 10.3389/fmed.2021.775587

**Published:** 2022-01-05

**Authors:** Dong Zhang, Hongcheng Han, Shaoyi Du, Longfei Zhu, Jing Yang, Xijing Wang, Lin Wang, Meifeng Xu

**Affiliations:** ^1^Institute of Artificial Intelligence and Robotics, Xi'an Jiaotong University, Xi'an, China; ^2^School of Automation Science and Engineering, Xi'an Jiaotong University, Xi'an, China; ^3^School of Software Engineering, Xi'an Jiaotong University, Xi'an, China; ^4^Dermatology Department, Second Affiliated Hospital of Xi'an Jiaotong University (Xibei Hospital), Xi'an, China; ^5^School of Information Science and Technology, Northwest University, Xi'an, China

**Keywords:** malignant melanoma, whole slide image, multi-scale feature, probability map, neural networks

## Abstract

Malignant melanoma (MM) recognition in whole-slide images (WSIs) is challenging due to the huge image size of billions of pixels and complex visual characteristics. We propose a novel automatic melanoma recognition method based on the multi-scale features and probability map, named MPMR. First, we introduce the idea of breaking up the WSI into patches to overcome the difficult-to-calculate problem of WSIs with huge sizes. Second, to obtain and visualize the recognition result of MM tissues in WSIs, a probability mapping method is proposed to generate the mask based on predicted categories, confidence probabilities, and location information of patches. Third, considering that the pathological features related to melanoma are at different scales, such as tissue, cell, and nucleus, and to enhance the representation of multi-scale features is important for melanoma recognition, we construct a multi-scale feature fusion architecture by additional branch paths and shortcut connections, which extracts the enriched lesion features from low-level features containing more detail information and high-level features containing more semantic information. Fourth, to improve the extraction feature of the irregular-shaped lesion and focus on essential features, we reconstructed the residual blocks by a deformable convolution and channel attention mechanism, which further reduces information redundancy and noisy features. The experimental results demonstrate that the proposed method outperforms the compared algorithms, and it has a potential for practical applications in clinical diagnosis.

## 1. Introduction

Malignant melanoma (MM) is a highly aggressive form of skin cancer whose incidence continues to increase at a great rate worldwide (1). It is characterized by an extraordinary metastasis capacity and chemotherapy resistance, and the difficulty of effective treatment increases with its continually developing aggression. Therefore, early diagnosis is essential to improve the survival rate of MM patients. Pathological examination is the gold standard for the diagnosis of MM (2), which enables the most reliable diagnosis based on pathological features at the cell level compared to other methods. Tissue cut from the lesion on the skin is made into pathological slices and scanned by a Digital Pathology Microscope Slide Scanner to get a whole-slide image (WSI). Through the WSI, the pathologist finds out the property of the tissue and marks the MM region, if it exists, to measure related pathological indicators, such as lesion size, invasion depth, etc., which provide an important reference for treatment planning and surgical prognosis (3).

Analyzing WSIs is a challenging task (4). Even an experienced pathologist spends an average of 10-20 min recognizing the region of MM in a WSI, of which identifying the MM region takes up much time. First, a WSI has billions of pixels, and the physician needs to perform a scanned screening of the pathology images in a zoomed-in window. Second, the complex visual characteristics of the skin lesions, such as irregular-shaped texture, fuzzy boundaries, etc., increase the difficulty of recognition. Some MM tissues are hard to distinguish from some benign tissues (5), which is a challenge for MM recognition. These problems aggravate the work burden of pathologists, affecting the efficiency of pathological examination. Third, the difficulty in training and scarcity of pathologists, as well as the uneven distribution of medical resources, make it difficult to obtain a timely and accurate diagnosis for every melanoma patient. Therefore, there is an urgent need for an effective method for automatic MM recognition in WSIs.

MM region screening in WSIs is an image recognition task that utilizes computer vision. Since convolutional neural networks (CNNs) have provided state-of-the-art image classification and segmentation performance, medical image analysis methods based on CNNs have been developed. The U-Net proposed by Ronneberger et al. (6) and its derivative improved networks (7–10) have achieved considerable success in medical image segmentation in recent years. However, pixel-wise image segmentation methods have limitations in MM region recognition in WSIs. The huge size of WSIs poses problems to the computation of the network. Some MM recognition methods based on deep learning are proposed. For example, Hekler et al. (11) trained a CNN based on ResNet-50 (12) to realize the classification of histopathological images of melanomas and nevi with an accuracy of 81%. The limited feature extraction capabilities of ResNet make it challenging to achieve higher accuracy. Wang et al. (13) used a deep CNN to establish a diagnosis model through the patch of eyelid melanoma histopathological slides and obtained good results. Yu et al. (14) proposed a method for melanoma recognition by leveraging very deep CNNs and constructed a fully convolutional residual network for accurate MM segmentation. However, it applies only to dermoscopy images analysis, which is easier to realize but not as reliable and detailed as pathological analysis.

However, these methods only work for the region of interest marked by pathologists. They cannot achieve good results in WSIs. The huge number of pixels makes network training difficult or impossible. Resizing images by down-sampling will lead to the loss of detailed information, which is unacceptable for MM diagnosis focusing on pathological features at the cellular level. Furthermore, due to the characteristics of WSIs and the limited feature extraction capability of related networks, the existing methods are difficult to adapt for WSIs-based MM recognition.

**Figure 1:**
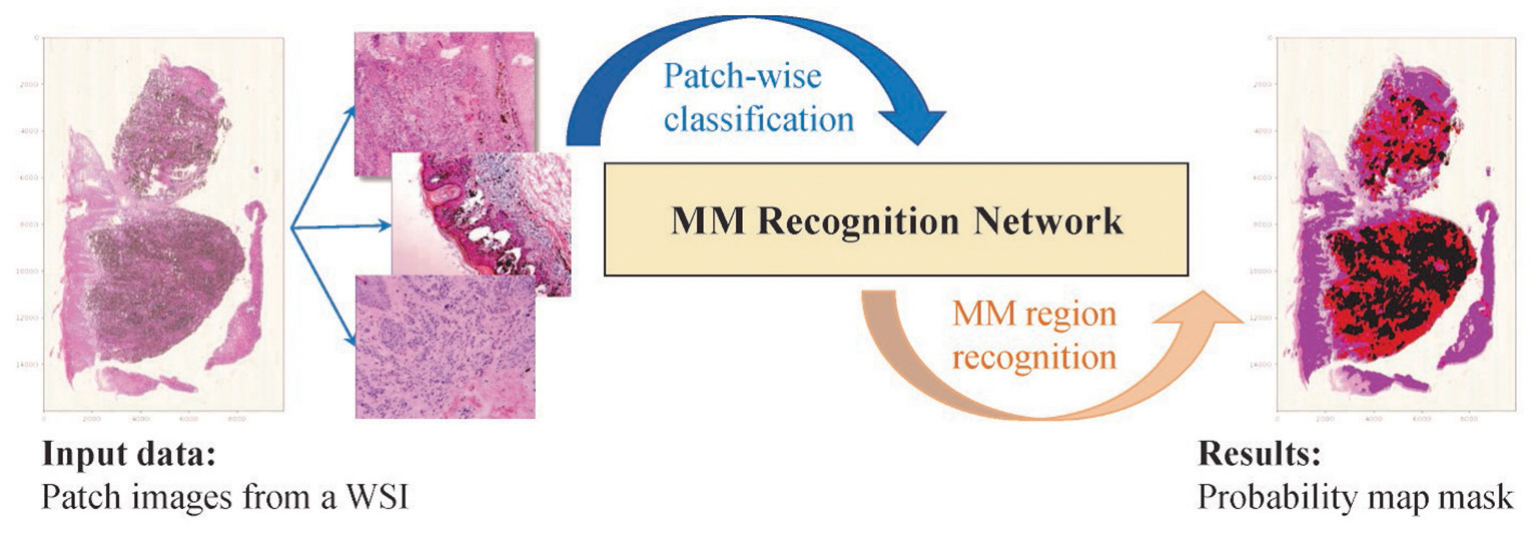
The idea of the proposed method. The WSI is broken up into patch images. The classification results are aggregated and generate the mosaic-type mask based on the probability map, and the MM regions are segmented based on a settable probability threshold.

Based on the above considerations, we proposed a novel MM recognition method based on a multi-scale feature representation and probability map to recognize the MM tissue region in WSIs, as shown in Figure 1. The following contributions are made to our work.

- Aiming at the difficult and inaccurate problems of recognizing the enormous size of WSIs, the breaking up the whole into parts idea is introduced to recognize melanoma. Furthermore, using predicted results and probabilities generates the mosaic-style mask and lesion region.
- To take both global and local features, we propose an efficient multi-scale network for improving melanoma recognition, combining high-level features with more semantic information and low-level features with more detail information. A multi-scale sliding cropping operation is used to obtain patch and sub-patch images.
- To enhance the feature representation of irregular-shaped lesions, highlight the critical features, and reduce the impact of information redundancy and data noise, we reconstruct the residual block by deformable convolution and channel attention.

The paper is arranged as follows. Section 2 details the proposed method, including the description of our method's framework, the realization principles, and the equations of each module. Section 3 shows the experimental results of our method, compares algorithms on an available WSI dataset, and further provides the ablation analysis to prove the effectiveness and rationality of the proposed method. Section 4 provides a further discussion on the feature representation capability of the proposed multi-scale network. And section 5 provides a brief summary and the conclusions of this work.

## 2. Methodology

### 2.1. Framework of Our Method

**Figure 2:**
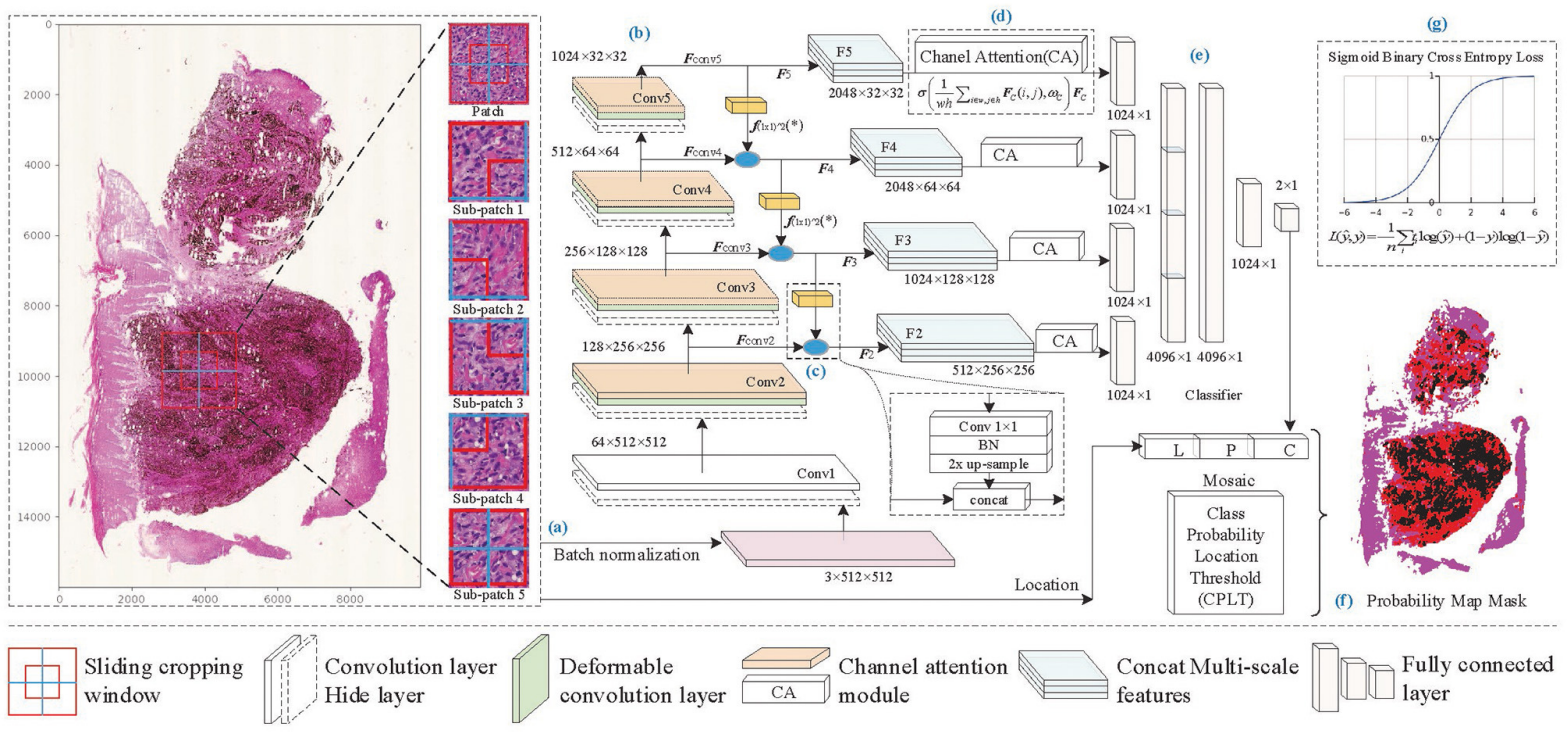
The framework of the proposed method. **(A)** Patch processing, **(B)** feature extraction, **(C)** feature fusion, **(D)** feature selection, **(E)** predictive classification, **(F)** probability map generation, and **(G)** loss function of training model.

On super-large WSI images, patch-based recognition is necessary and feasible. Melanoma pathological analysis mainly focuses on cell-scale characteristics. We set patch size according to pathologists' professional advice, which ensures that cell morphology and local distribution are well represented in the patches. On the boundary of the patch, some cells may be torn, but the overlapping sampling method can effectively avoid the loss of information caused by incomplete splitting. For lesion areas without clear boundaries, mixed cell tissue limits feature extraction by conventional rectangular convolution. Therefore, the proposed method reconstructed the residual block by deformable convolution and channel attention to overcome the irregular-shaped lesion and focus on important features. Furthermore, to overcome the influence of cell-scale differences, we built multi-scale feature fusion layers to enhance feature information and improve identification accuracy. The framework of the proposed method shown in Figure 2 consists of the following seven components: patch processing, feature extraction, feature fusion, feature selection, predictive classification, mask generation, and loss function in training.

- **Patch processing**: A WSI is broken up into *N* patches through sliding cropping (*N* depends on the sliding window size and sliding stride), from which tissue-contained patches are picked out by a color analysis method. Each tissue-contained patch is broken up into sub-patches. And then, patch images and sub-patch images are normalized to a uniform size.
- **Feature extraction**: The lesion features are extracted by backbone Conv1 to Conv5. Considering the irregular-shaped cells, and focusing on essential features, deformable convolution (DC) and channel attention (CA) operations are embedded in the Conv2 to Conv5 layers to enhance the feature extraction capability of the network. Then extracted features ***F***_*convi*_(*i* = 2, 3, 4, 5) are produced separately from Conv2 to Conv5.
- **Feature fusion**: As the network is gradually deepened, the resolution of the feature map decreases, and the semantic properties of the features are enhanced. The features of the next layer, which contains richer semantic information, are concatenated with those of the current layer, which contains richer detailed texture information, to enhance the lesion feature representation capability of the network.
- **Feature selection**: After the fused features ***F***_*i*_(*i* = 2, 3, 4, 5), the channel attention mechanism is separately used to select the critical features and to enhance the correlation between high-level semantic features and low-level detailed features.
- **Predictive classification**: The output features from each branch are flattened into a vector, respectively, and then they are concatenated. Fully connected layers are constructed to obtain the predictive classification results of patch images.
- **Probability map generation**: The prediction results of patches (containing prediction labels and confidence probabilities) are combined with the location information to generate the probability map of malignant tissues. And a mosaic-type mask of MM regions is obtained through a confidence probability threshold.
- **Loss function**: Sigmoid binary cross-entropy loss function is used in training for parameter optimization.

### 2.2. Multi-Scale Features

In the pathological examination, it is necessary to carry out comprehensive analysis according to various characteristics of lesions, such as tissue morphology and cell distribution, which are reflected on a large scale, and cell morphology and nuclear size, which are reflected on a smaller scale (15). Therefore, computational analysis of WSIs at different scales is beneficial to represent pathological features at different scales. A multi-scale sliding cropping method is embedded in the proposed algorithm. For each patch of a WSI, besides the whole patch image, cropped sub-patch images from the patch are normalized to a uniform size and input into the network together for the classification of the patch. The size and quantity of the sub-patch depend on the cropping method, for example, Figure 2A shows five sub-patches cropped from a patch image.

Furthermore, the idea of multi-scale is also reflected in the construction of the feature extraction network. As the network deepens, feature resolution decreases and channels increase, low-level detail information is being transformed into higher-level semantic information. However, factors such as data noise and chain derivative attenuate or lose the information in the forward and back propagation, which becomes more and more apparent with increasing network depth. The fusion of shallow features and deeper features, which are with different scales, to supplement the semantic information of high-level features is beneficial to improve the feature representation capability of the network. Based on the above considerations, a network with enhanced multi-scale feature extraction capability is constructed. As Figure 2 shows, additional branches are added to the backbone network for feature fusion. In each branch, the feature of (*i* + 1)-th level ***F***_Conv*i*+1_(*i* = 2, 3, 4) is concatenated with the feature of *i*-th level ***F***_Conv*i*_(*i* = 2, 3, 4) after 1 × 1 convolution and up-sampling, as shown in Figure 2C, and then ***F***_*i*_(*i* = 2, 3, 4) is obtained, as shown in Equation (1).


(1) $$\begin{array}{l} F_{i}=f_{\left(1\times 1\right)*2}\left(F_{i+1}\right)⊕F_{\text{Conv}i} \end{array}$$

where *f*_(1×1)*2_(*) indicates that the ***F***_*i*_ is obtained by the convolution of 1 × 1 and double up-sampling of features ***F***_*i*+1_ and has the same shape maps as the *i*-th conv output features ***F***_Conv*i*_. ⊕ denotes the concatenation of the normalized features of the two groups.

The concatenated features ***F***_2_ ~ ***F***_4_, together with ***F***_5_, which is obtained from ***F***_Conv5_, are transferred to feature vectors and input into the fully connected layer via shortcut connections for classification.

### 2.3. Deformable Convolution

**Figure 3:**
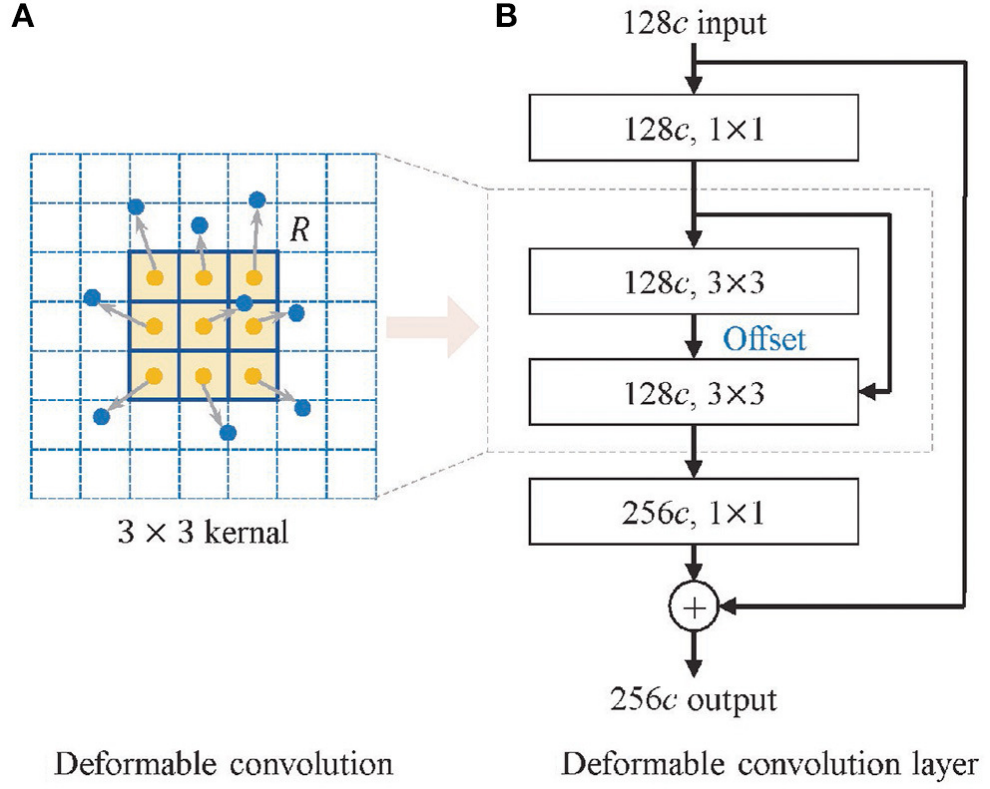
Structure of deformable convolution module. **(A)** The deformable convolution and **(B)** the deformable convolution layer.

MM tissues in histopathological images mainly show as interstitial or heterogeneous tumor cells, which are mainly enlarged and darkly stained nuclei with varying shapes (16). This irregularity leads to the inadequate learning of melanoma feature information by traditional convolution for its fixed rectangular receptive field of the kernel. Inspired by Dai et al. (17) and Zhu et al. (18), we introduce offsets in the traditional convolution to make the geometry of the kernel more flexible, as shown in Figure 3, which improves the representation of irregular-shaped features. The deformable convolution format for each position *p* in the input feature map is shown in Equation (2).


(2) $$\begin{array}{l} y\left(p\right)=\underset{p_{k}\in R}{\sum }w\left(p_{k}\right)\cdot x\left(p+p_{k}+\Delta p_{k}\right) \end{array}$$

where ***y***(*p*) indicates the feature obtained by the convolution on one sampling point *p* of the feature map. ***R*** is the receptive field size of the regular kernel. *p*_*k*_ denotes the difference between the sampling points and ***y***(*p*), *k* = 1, 2, 3…*N, N* = |***R***|, Δ*p*_*k*_ is the learned offset, and ***w*** is the kernel parameter. The offset of the deformable convolution has a dilated value, which determines the maximum distance for resampling and is set to 2.

### 2.4. Channel Attention

First, multi-scale feature fusion enriches the extracted feature information of the network, but while enhancing feature representation capability, it also brings some redundant features, which are unrelated to melanoma recognition, and interferes with model learning. It is particularly obvious in low-level features with higher resolution, and this effect becomes more prominent when low-level features are fused with high-level features through additional branch paths. Second, the deformable convolution helps in the feature extraction of irregular lesions and enhances lesion feature representations, but also generates some noisy features influenced by non-lesion tissue. Therefore, to extract more valuable information and suppress the impact of redundant information and noises features, we need a mechanism that focuses on essential features and filters the irrelevant features.

**Figure 4:**
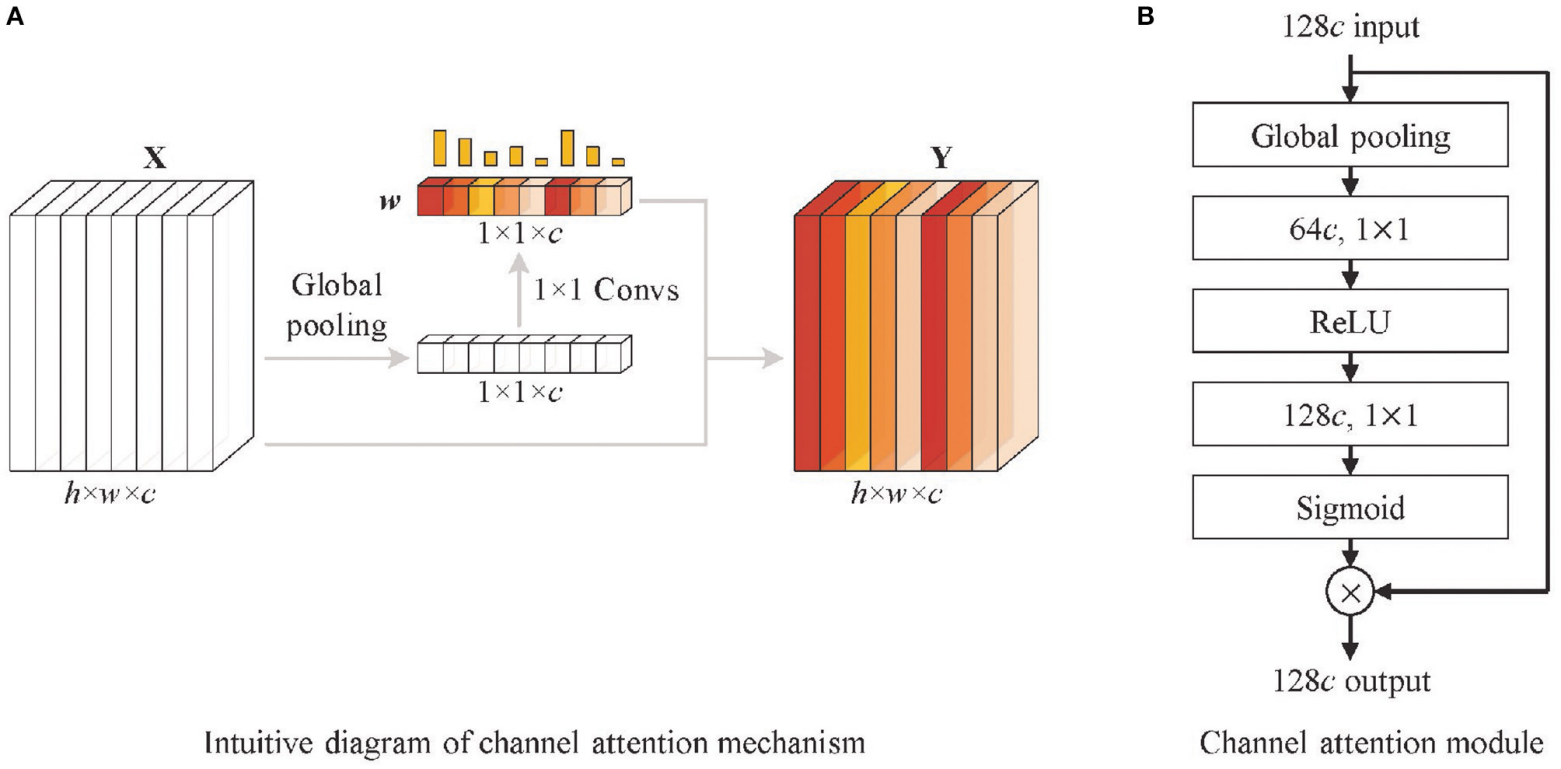
Structure of channel attention module. **(A)** The intuitive diagram of the channel attention mechanism and **(B)** channel attention module. It firstly performs global pooling to the input feature map (128c denotes 128 channels) to obtain the overall representations. Then a weight vector is learned by 1 × 1 convolution operations. The learned weight vector allocates weight coefficients for each channel of the original feature map, and the weighted feature map is output.

Based on the above considerations, and inspired by the work of Hu et al. (19), a channel attention mechanism is used in the shortcut connection between features ***F***_*i*_(*i* = 2, 3, 4, 5) and fully connected layers, as shown in Figure 2D. It highlights high-value feature maps by a series of weights learned by the channel attention (CA) module. The filtering of channels is actually the weighting of different types of features. Although the convolution operation itself also correlates each channel of the feature map with each other, it is difficult to accurately assign appropriate weights to each channel due to the influence of the *w* and *h* dimensional feature distributions. To address this problem, the channel attention mechanism obtains a global representation of each channel by global pooling, and the weights of each channel are calculated by 1 × 1 convolution based on the resulting feature vectors. In Figure 4, the CA module firstly performs global pooling to the input feature map to obtain the overall representation of it. Then a weight vector is learned by 1 × 1 convolution. The learned weight vector allocates weight coefficients for each channel of the original feature map, and the weighted feature map is output. The mathematic description of the channel attention module is formatted as Equation (3).


(3) $$\begin{array}{l} Y=\sigma \left(W_{Conv2}\delta \left(W_{Conv1}\frac{1}{hw}\underset{i\in h,j\in w}{\sum }X\left(i,j\right)\right)\right)⊗X \end{array}$$

where ***X*** means the input feature map, and ***Y*** denotes the output feature map of the channel attention module, *h* and *w* are the height and width in the input feature maps. ***W***_*Conv*1_ and ***W***_*Conv*2_ indicate the parameters of two 1 × 1 convolution operations, which are equivalent operations to fully connected layers. ***δ*** is the ReLU activation. ***σ*** is the sigmoid function, and ⊗ means the weighting calculation of the learned weight vector and the input feature map.

In addition to calculating the channel weights of feature weights, the channel attention mechanism also strengthens the correlation between channels through global pooling and 1 x 1 convolution; making up for the defect of the weak correlation between channels in the convolution module is conducive to the enhancement of feature expression ability. Therefore, the channel attention modules are also embedded into the backbone network, as shown in Figure 2B.

## 3. Experiments

### 3.1. Experimental Setup

MM WSIs labeled by pathologists are rare and valuable data. The dataset is collected from the Second Affiliated Hospital of Xi'an Jiaotong University (Xibei Hospital), containing 30 WSIs labeled by experienced pathologists. Sliding window size is set to 1, 024 × 1, 024, sliding stride is set to 1024, 18,698 tissue-included patches are obtained, containing 7,369 malignant tissue patches and 11,329 benign tissue patches. They are divided into training, validation, and test datasets by a ratio of 6:2:2. Five sub-patches are cropped from each patch, as shown in Figure 2A, and all images are resized to 512 × 512 when input to the network. Considering that melanoma tissue features are non-directional and non-chiral, we introduce data augmentation operations by the mirror and random rotation in the range of (−90°, +90°).

The proposed method is developed by Python 3.6 on Ubuntu18.04, and the hardware is RTX2080-12G with CUDA-10.1. The development libraries include MXNet-1.5, Gluoncv-0.5, Numpy-1.17, OpenCV-4.2, etc. The models iterate 30 epochs, and the batch size is 32. Gradient descent with momentum (20) is used for optimization. We set the momentum to 0.9. The learning rate is 0.001, and the decay rate is 0.99. Both recall (*R*) and precision (*P*) for MM recognition are considered in diagnosis, so the evaluation criterion *F*1 score is used to comprehensively measure the performance of the proposed method, which is calculated as Equation (4).


(4) $$\begin{array}{l} F1=\frac{2\times P\times R}{P+R} \end{array}$$

### 3.2. Results of Patch Classification

In order to verify the effectiveness and recognition performance of the algorithm, the proposed method is compared with some popular algorithms in recent years, including Inception V3 (21), ResNeXt (22), SENet (19), and ResNeSt (23). The experimental results are shown in Table 1, the higher the values of *F*1, recall, precision, and accuracy, the better the recognition performance. The *F*1 values of all algorithms exceeded 90%, except Inception V3, and the scores of the proposed method also achieved the best results. SENet and ResNeSt, containing the channel attention module, outperform other comparison algorithms, indicating that the channel attention mechanism improves performance.

**Table 1:** The experimental results of the proposed algorithms and comparison algorithms, the higher the values of precision, recall, accuracy, and *F*1, the better the recognition performance.

**Algorithms**	**Layer**	**Precision**	**Recall**	**Accuracy**	***F*1**
Inception V3 (2016)	50	0.8919	0.8876	0.8326	0.8897
ResNeXt (2017)	50	0.8974	0.9241	0.8618	0.9106
SENet (2018)	50	0.8915	0.9472	0.8721	0.9185
SENet (2018)	101	0.9120	0.9477	0.8812	0.9295
ResNeSt (2020)	50	0.9314	0.9327	0.8877	0.9321
ResNeSt (2020)	101	0.9526	0.9601	0.9355	0.9513
MPMR (ours)	50	0.9683	0.9709	0.9498	0.9696
**MPMR (ours)**	101	**0.9740**	**0.9861**	**0.9553**	**0.9749**

*Bold indicate maximum values*.

The proposed method outperforms all the comparison algorithms for the same number of layers, mainly benefiting from the deformable convolution, the channel attention, and the multi-scale feature fusion. In particular, the learning capability of multi-scale features in the proposed method effectively adapts the different scale samples. It sufficiently learns the feature information of melanoma in the training and validation datasets and has better robustness on the testing dataset. Therefore, the proposed algorithm outperforms other algorithms on the WSI test dataset.

### 3.3. Results of the Probability Map

**Figure 5:**
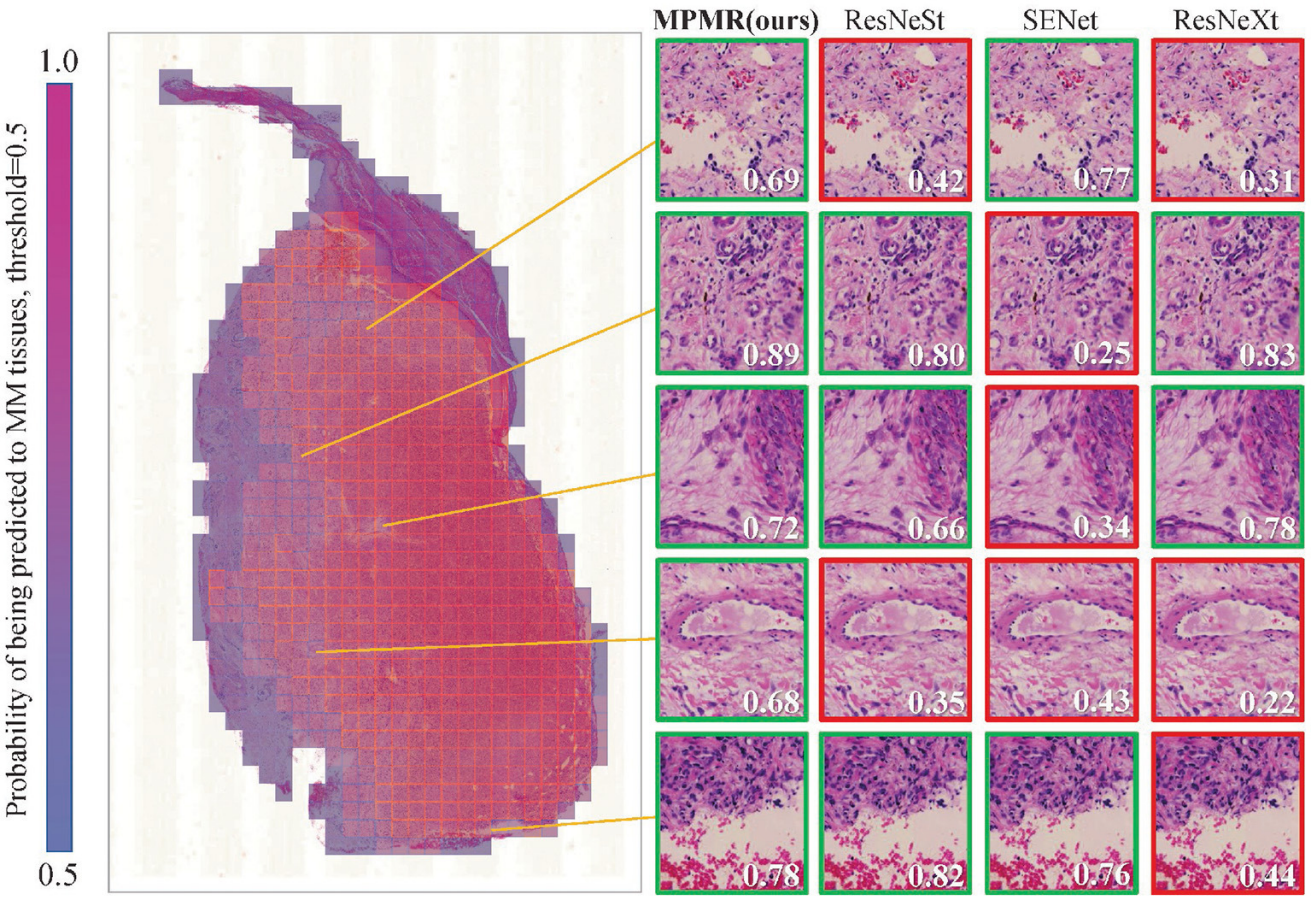
The mosaic-style mask result of a WSI in the test dataset is generated through the probability map obtained through patch image classification. The recognition results of some patches, which are difficult to recognize, through different algorithms are compared. A green box represents a correct prediction, and a red box represents a misclassification. The probabilities of being predicted as MM tissues are marked in each patch image, and the threshold is 0.5.

The prediction results of patch images containing prediction labels and confidence probabilities are combined with the location information to generate the probability map. The visualization results of a WSI containing malignant melanoma tissues are shown in Figure 5. The probability of being predicted as MM tissues is visualized as different colors, from 0 to 1. The threshold of malignant tissues and benign tissues is set to 0.5; red regions display the recognized MM tissues. The prediction results of some difficult samples of different algorithms are compared, and the proposed method provides the most correctly recognized patches, marked by green boxes, while other comparison algorithms provide some incorrect recognition results, marked by red boxes. The results indicate that the proposed method can obtain more accurate recognition results in WSIs.

### 3.4. Ablation Analyses

To analyze the contributions of multi-scale feature fusion, deformable convolution, and channel attention in the proposed method, ablation analyses are performed for these impacts. The results of ablation analyses are shown in Tables 2–4, the higher the values of precision, recall, accuracy, and *F*1, the better the recognition performance.

**Table 2:** The experimental results of the ablation analysis of multi-scale features, the higher the values of precision, recall, accuracy, and *F*1, the better the recognition performance.

**Multi-scale features**	**Layer**	**Precision**	**Recall**	**Accuracy**	***F*1**
F5	50	0.9400	0.9457	0.9367	0.9428
F5/F4	50	0.9448	0.9633	0.9493	0.9540
F5/F4/F3	50	0.9590	0.9675	**0.9567**	0.9632
F5/F4/F3/F2	50	**0.9683**	**0.9709**	0.9498	**0.9696**

*Bold indicate maximum values*.

**Table 3:** The experimental results of the ablation analysis of deformable convolution, the higher the values of precision, recall, accuracy, and *F*1, the better the recognition performance.

**Deformable convolution**	**Layer**	**Precision**	**Recall**	**Accuracy**	***F*1**
None	50	0.9380	0.9509	0.9387	0.9444
DConv5	50	0.9396	0.9522	0.9402	0.9458
DConv5/4	50	0.9464	0.9563	0.9462	0.9513
DConv5/4/3	50	0.9604	0.9619	**0.9569**	0.9611
DConv5/4/3/2	50	**0.9683**	**0.9709**	0.9498	**0.9696**

*Bold indicate maximum values*.

**Table 4:** The experimental results of the ablation analysis of channel attention, the higher the values of precision, recall, accuracy, and *F*1, the better the recognition performance.

**Channel attention**	**Layer**	**Precision**	**Recall**	**Accuracy**	***F*1**
None	50	0.9320	0.9216	0.9222	0.9242
Backbone (B)	50	0.9340	0.9321	0.9256	0.9331
Shortcut (S)	50	0.9472	0.9552	0.9460	0.9512
Both B and S	50	**0.9683**	**0.9709**	**0.9498**	**0.9696**

*Bold indicate maximum values*.

#### 3.4.1. Multi-Scale Features

The proposed method realizes multi-scale feature fusion by constructing additional branch paths and adopting shortcut connections between fused features and the fully connected layers. Low-level features containing more detail information are expected to supplement the semantic features of high-level features for enhancing the classification capability of the model. The experimental results of the networks with different numbers of branch paths are shown in Table 2. The more branch paths added, the higher the values of *F*1, recall, precision, and accuracy obtained. It indicates that the prediction method based on multi-scale features helps the proposed method to recognize melanoma. In the results of F5/F4/F3/F2, the accuracy of the proposed method decreases compared to F5/F4/F3, and the other evaluation indicators show weak increases. It indicates that multi-scale feature fusion should be carried out in an appropriate range, and an excess of fusions will cause information redundancy, which is not conducive to feature representation.

#### 3.4.2. Deformable Convolution

The melanoma characteristics in the pathological images are mainly enlarged and darkly stained nuclei with varying shapes. This irregularity leads to the inadequate learning of melanoma feature information by traditional convolution. Deformable convolution is embedded into the convolution layers of the proposed network to enhance irregular-shaped feature representation ability. The experimental results of the networks with different numbers of deformable convolution layers are shown in Table 3, indicating that the more deformable convolution layers embedded, the better the recognition performance of the proposed method. Accuracy decrease occurs in the results of DConv5/4/3/2. It indicates that too many deformable convolution layers may amplify the impact of noise on learning and influence feature representation.

#### 3.4.3. Channel Attention

Channel attention in the proposed method selectively enhances information-rich features, allowing subsequent processing of the networks to take full advantage of these features and suppress noisy features. The experimental results of channel attention with different numbers of layers are shown in Table 4, where the B-case indicates that channel attention modules are only embedded in the backbone network for feature extraction, and the S-case indicates that channel attention modules are used in the shortcut connection between fused features and fully connected layers. The recognition performance of the model is significantly improved after using channel attention modules. However, both the embedded B-case and S-case can obtain the best performance of the proposed method. This further demonstrates that the embedding of channel attention can facilitate positive network learning.

## 4. Discussion

The pathological features related to melanoma are at different scales, such as tissue, cell, and nucleus, and enhancing the representation of multi-scale features is important for melanoma recognition. From the experimental results, it can be concluded that the residual block based on deformable convolution and multi-scale feature fusion brings considerable performance improvement in the patch-wise classification of WSIs.

The visual features of malignant melanoma and benign nevi tissues are very similar, and the shape of the features is irregular and has uneven distribution, which further increases the difficulty of recognition. When learning the feature space of a histopathological skin image, the traditional convolutional network is limited by its fixed spatial geometric structure, as shown in Figure 3, which is not suitable for the irregular shape of lesions and the uneven distribution of melanoma cells. However, the deformable convolution layers effectively avoid the rectangular limitation of traditional convolution sampling. The experimental results in Table 3 demonstrate that dynamic convolution can better extract the features of tissue images. The performance improvement of deformable convolution on the model grows as the number of layers increases, which introduces extra computational consumption but can be neglected for some tasks with low real-time requirements.

Shortcut connections, also known as skip connections, show considerable advantages in residual networks and U-shaped networks. The residual connections ensemble the feature at different layers through sum operation, and (24) put forward similar views. The connections between the encoder and the decoder in U-shaped networks, through deconvolution and concatenation, realize the fusion of features at different scales. In addition, extra information flows, brought by shortcut connections, provide shorter paths for the transmission of parameters in the forward- and back-propagation, reducing information attenuation. These ideas are embodied in the construction of the proposed networks. The additional branch paths in the proposed network realize multi-scale feature fusion through 1 × 1 convolution, 2× up-sampling, and concatenation. Another fusion of several fused features is performed through the shortcut connections between the fused features and the fully connected layers. The above operations are expected to enhance feature representation and make contributions to improve MM recognition precision.

**Figure 6:**
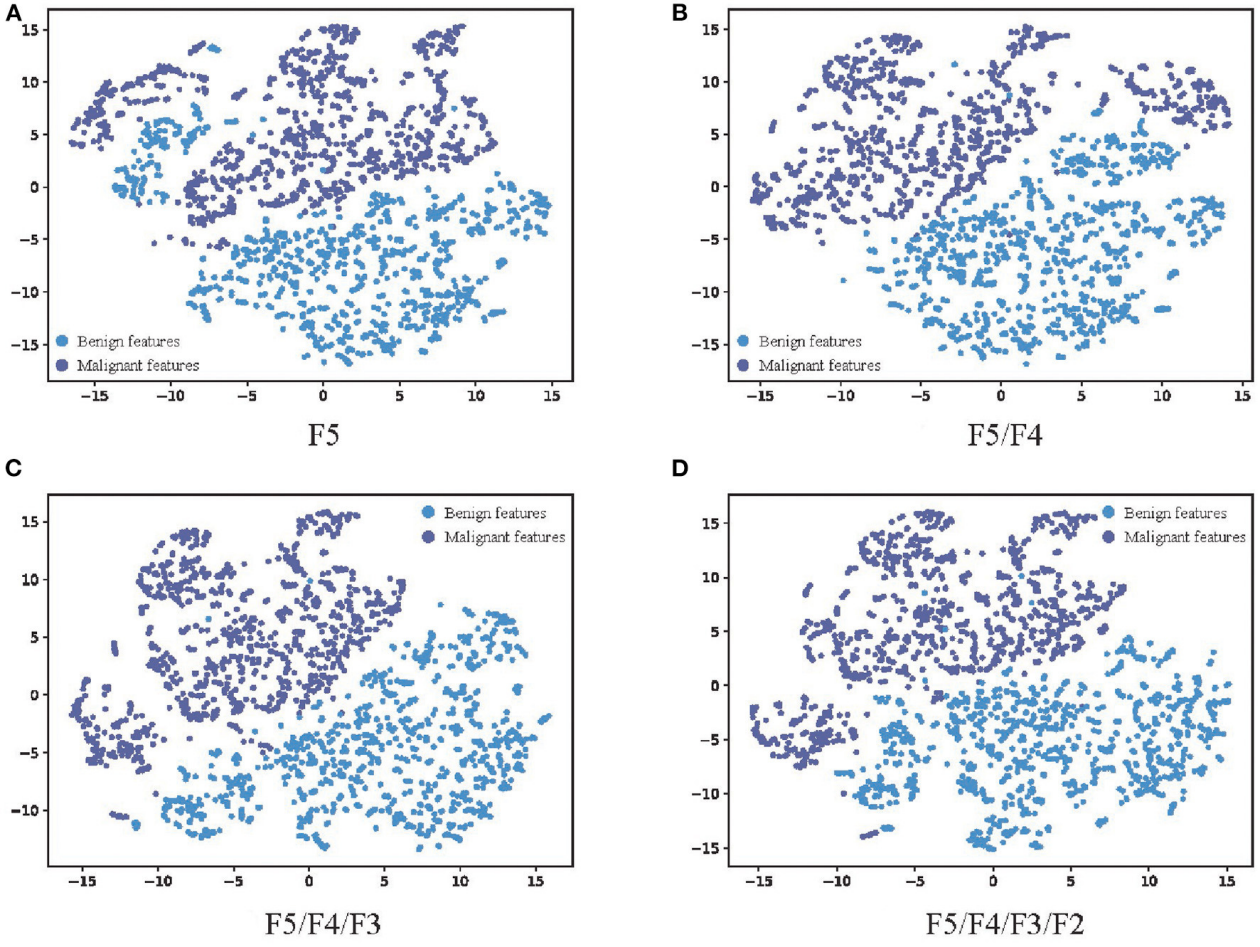
The visualization results of the features extracted by networks with different numbers of feature fusion branch paths. **(A)** F5, **(B)** F5/F4, **(C)** F5/F4/F3, and **(D)** F5/F4/F3/F2. The feature vector in the second-to-last layer of the full connection layers are transferred from 1,024 dimensions to 2 dimensions through t-SNE. The blue scatter plots represent malignant features, and the purple scatter plots represent malignant features. The higher the linear separability of benign features and malignant features after dimensionality reduction, the more beneficial the features extracted from the network are for classification.

In order to further analyze the influence of multi-scale fusion on the quality of features extracted from the network, t-distributed stochastic neighbor embedding (t-SNE) (25), a manifold learning dimensionality reduction method, is used to visualize the features extracted from the network with the different number of feature fusion branch paths. The feature vectors in the second-to-last layer of the full connection layers are transferred from 1,024 dimensions to 2 dimensions and visualized as shown in Figure 6. The higher the linear separability of benign features and malignant features after dimensionality reduction, the more beneficial the features extracted from the network are for classification. The dimension reduction results of F5, which represents the features extracted by the network without multi-scale feature fusion, are shown in Figure 6A, and some of the benign features are interspersed with the malignant features. The results of F5/F4 shown in Figure 6B, which represents the features extracted by the network with one branch path for multi-scale feature fusion, show considerable improvement. Figures 6C,D show more improvements, which indicates that the additional branch paths for multi-scale feature fusion improve the quality of the features extracted by the network, enhancing the feature representation for MM recognition, and finally provide more accurate MM recognition results. This is consistent with the experimental results in Table 2.

## 5. Conclusions

This work proposes a novel automatic MM recognition method in WSI based on multi-scale features and the probability map. The idea that breaking up a WSI into patches and sub-patches through multi-scale sliding cropping solves the difficult-to-calculate problem of WSIs with huge sizes, and the probability map is generated based on the predicted class and confidence probabilities and location information of patch images to visualize the recognition result of MM tissues in WSIs. Additional branch paths and shortcut connections are established for multi-scale feature fusion, which realizes the information supplement of low-level features containing more detail information to deep features containing more semantic information. Deformable convolution operations are embedded into the backbone network to enhance the representation capability of irregular-shaped features in tissues. Channel attention modules are used in the shortcut connection between fused features and fully connected layers, and also the backbone network to highlight the high-value features and reduce the negative impacts of information redundancy caused by additional branch paths.

The results of comparison experiments indicate that the proposed method outperforms Inception V3, ResNeXt, SENet, and ResNeSt. The results of ablation analyses prove the effectiveness of multi-scale feature fusion, deformable convolution, and channel attention modules. Through the proposed method, MM regions in WSIs can be recognized accurately and efficiently, which is a great help to pathological examination and the diagnosis of MM.

## Data Availability Statement

The raw data supporting the conclusions of this article will be made available by the authors, without undue reservation.

## Author Contributions

DZ and HH were in charge of experiments and manuscript writing. LZ and MX were responsible for medical analysis and annotation of pathological data. SD and JY provided guidance for method formulation. LW and XW checked the experimental results. All authors contributed to the article and approved the submitted version.

## Funding

This work was supported by the National Key Research and Development Program of China under (grant no. 2017YFA0700800), the National Natural Science Foundation of China under (grant no. 61971343), the Shaanxi Provincial Social Science Fund under (grant no. 2021K014), and the Key Research and Development Program of Shaanxi Province of China under (grant no. 2020GXLH-Y-008).

## Conflict of Interest

The authors declare that the research was conducted in the absence of any commercial or financial relationships that could be construed as a potential conflict of interest.

## Publisher's Note

All claims expressed in this article are solely those of the authors and do not necessarily represent those of their affiliated organizations, or those of the publisher, the editors and the reviewers. Any product that may be evaluated in this article, or claim that may be made by its manufacturer, is not guaranteed or endorsed by the publisher.
